# 

**Published:** 1869-04-01

**Authors:** 


### *CONTENTS.*

TRANSLATIONS:

Pathogenesis ........ 193

CORRESPONDENCE ........

The Waukesha Medicinal Spring ........ 203

ORIGINAL COMMUNICATIONS:

Tubercular Kidneys ........ 208

EDITORIAL: ........ 210

OBITUARY:

Grisolle—Dunn ........ 212

FOREIGN ITEMS:

Medical Education in Spain ........ 213

Germany—Colloid of the Brain ........ 215

Upon the Mode of Action of a Group of Poisons ........ 216

Animal Heat ........ 218

Investigations into the Toxicological Properties of Aconitine ........ 219

LOOT:

Pepsine ........ 221

Pigeons and Opium—Hall's Opinion of Stricture—Medicated Pessaries—Syr. Ferri Iodid ........ 222

Styptic Paper—Gonorrhæ—Chlorea ........ 223

Poisonous Red Dyes—Antidote for Strichnia—Tendo Achillis ........ 225

